# *Jacalin-related lectin 45* (*OsJRL45*) isolated from ‘sea rice 86’ enhances rice salt tolerance at the seedling and reproductive stages

**DOI:** 10.1186/s12870-023-04533-z

**Published:** 2023-11-09

**Authors:** Qinmei Gao, Xiaolin Yin, Feng Wang, Congzhi Zhang, Feicui Xiao, Hongyan Wang, Shuchang Hu, Weihao Liu, Shiqi Zhou, Liangbi Chen, Xiaojun Dai, Manzhong Liang

**Affiliations:** 1 Hunan Province Key Laboratory of Crop Sterile Germplasm Resource Innovation and Application, College of Life Science, Hunan Normal University, Changsha, 410081 China; 2 College of Chemistry and Chemical Engineering, Jishou University, Hunan, 416000 China

**Keywords:** Rice (*Oryza sativa* L.), Salt tolerance, *OsJRL45*, Na^+^/K^+^ homeostasis

## Abstract

**Background:**

Rice (*Oryza sativa* L.) is one of the most widely cultivated grain crops in the world that meets the caloric needs of more than half the world’s population. Salt stress seriously affects rice production and threatens food security. Therefore, mining salt tolerance genes in salt-tolerant germplasm and elucidating their molecular mechanisms in rice are necessary for the breeding of salt tolerant cultivars.

**Results:**

In this study, a salt stress-responsive jacalin-related lectin (*JRL*) family gene, *OsJRL45*, was identified in the salt-tolerant rice variety ‘sea rice 86’ (SR86). *OsJRL45* showed high expression level in leaves, and the corresponding protein mainly localized to the endoplasmic reticulum. The knockout mutant and overexpression lines of *OsJRL45* revealed that *OsJRL45* positively regulates the salt tolerance of rice plants at all growth stages. Compared with the wild type (WT), the *OsJRL45* overexpression lines showed greater salt tolerance at the reproductive stage, and significantly higher seed setting rate and 1,000-grain weight. Moreover, *OsJRL45* expression significantly improved the salt-resistant ability and yield of a salt-sensitive *indica* cultivar, L6-23. Furthermore, *OsJRL45* enhanced the antioxidant capacity of rice plants and facilitated the maintenance of Na^+^-K^+^ homeostasis under salt stress conditions. Five proteins associated with OsJRL45 were screened by transcriptome and interaction network analysis, of which one, the transmembrane transporter Os10g0210500 affects the salt tolerance of rice by regulating ion transport-, salt stress-, and hormone-responsive proteins.

**Conclusions:**

The *OsJRL45* gene isolated from SR86 positively regulated the salt tolerance of rice plants at all growth stages, and significantly increased the yield of salt-sensitive rice cultivar under NaCl treatment. *OsJRL45* increased the activity of antioxidant enzyme of rice and regulated Na^+^/K^+^ dynamic equilibrium under salinity conditions. Our data suggest that *OsJRL45* may improve the salt tolerance of rice by mediating the expression of ion transport-, salt stress response-, and hormone response-related genes.

**Supplementary Information:**

The online version contains supplementary material available at 10.1186/s12870-023-04533-z.

## Background

Rice (*Oryza sativa* L.) is one of the main food crops in the world, and the demand for rice continues to increase rapidly with population growth [1]. However, soil salinization poses a serious threat to agricultural production and food security. Rice is sensitive to high salinity [2, 3] and is easily affected by salt stress at all developmental stages [4], which eventually leads to a severe reduction in yield and quality [5–7]. Therefore, to guarantee food safety and security, it is important to enhance the salt tolerance of rice and make full use of the land resources.

Salt stress can cause osmotic imbalance, ionic toxicity, and nutrient depletion, which in turn can cause the accumulation of harmful substances, decreased photosynthetic benefits, reduced yield, and even plant death [8]. High salinity reduces the content of small organic molecules and soluble sugars in rice, causing an infiltration effect [9]. Excessive accumulation of reactive oxygen species (ROS) in cells leads to increased membrane permeability, causing membrane lipid peroxidation, protein damage, and cell structure destruction and/or cell death [10]. Intracellular Na^+^-K^+^ homeostasis is critical for the survival of rice plants under salt stress, and the accumulation of excess Na^+^ in plant cells under high salinity causes ionic poisoning and steady state imbalance, leading to metabolic disorders [11–13]. This ultimately leads to plant death because excessive salt directly affects cellular metabolism and gas exchange in rice [14].

To enhance the ability of rice plants to resist salt stress, extensive research has been conducted to interpret the molecular mechanisms of salt tolerance, and considerable progress has been made [15, 16]. For example, *terpene synthase* (*TPS*) family genes increase the content of trehalose and other organic substances, under high salinity conditions to enhance the salt tolerance of rice [17, 18]. The *ascorbate peroxidase* (*APX*) family genes [19–21] and some transcription factors [22, 23] are involved in maintaining the stability of the antioxidant system to improve the salt stress tolerance of rice. Other gene families such as *HKT*, *NHX*, and *HAK* play an important role in regulating the Na^+^-K^+^ balance [24–27]. Quantitative trait locus (QTL) mapping revealed hundreds of potential salt stress-responsive genes [28–31]. Some candidate genes have also been replicated and functionally validated. For example, *SKC1* increases K^+^ levels under high salinity to regulate salt tolerance [32], *DST* regulates hydrogen peroxide (H_2_O_2_) balance and reduces Na^+^ inflow to increase salt stress tolerance [33], and *OsGATA8* increases salt tolerance by reducing the Na^+^-K^+^ content [34]. However, most of this research has focused on the mechanisms of salt tolerance in rice at the seedling period, not on the salt tolerance mechanism during the reproductive period, which is generally considered to be governed by different genes [35]. Therefore, it is important to excavate key salt stress-responsive genes and elucidate the salt tolerance mechanisms of rice at different stages for the selection and breeding of salt-tolerant varieties.

‘Sea rice 86’ (SR86) is a new rice cultivar domesticated from a wild strain first found submerged in sea water in 1986 [36]. Preliminary studies show that SR86 exhibits strong salt tolerance when grown in the presence of 0.9% salt [37], and maintains osmotic regulation and antioxidant capacity under high salt stress [38]. RNA sequence analysis of SR86 found a number of salt-induced genes [29]. Genome-wide association study (GWAS) revealed 51 genomic regions involved in salt stress [39]. A QTL mapping study revealed that qST1.1 may be related to the salt tolerance of SR86 [40]. However, only a few of the salt tolerance-related genes identified in SR86 have been functionally characterized to date.

*Jacalin-related lectin* (*JRL*) genes have been found in many different plant species, and their functions have attracted much attention in recent years [41]. JRLs have a mannose-binding lectin domain and can form complexes with glycoproteins for endogenous regulation [42] and abiotic stress responses [43]. Zhang et al. found that salt could induce an increase in the transcription level of the *JRL* gene in rice [44]. In recent years, other plant species including wheat (*Triticum aestivum*) [45], poplar (*Populus euphratica*) [46], and barley (*Hordeum vulgare*) [47] have achieved similar results, these results indicate that *JRL* family genes play an important role in salinity stress response. The rice genome contains 28 *JRL* genes [48], two of which were reported to respond to salt stress [49, 50]. However, whether other rice *JRL* genes respond to salt stress and the mechanism of salt stress response remain unknown.

In this study, we identified a salt stress-related *JRL* gene in SR86 by bulked segregant analysis and sequencing (BSA-seq), and named it *OsJRL45*. GUS staining and subcellular localization analyses were conducted to elucidate its spatial expression profile. A genetics approach was used to clarify its role in salt tolerance. The physiological and molecular mechanisms of salt tolerance of OsJRL45 were elucidated by analyzing its antioxidant activity and ion homeostasis. Transcriptome analysis, weighted gene co-expression network analysis (WGCNA), and String interaction network analysis were performed to elucidate key genes and regulatory pathways of salt tolerance. Overall, the results of this study provide key insights, which could be used to further explore the mechanism of plant salt tolerance.

## Results

### Mapping of salt tolerance-related genes in SR86

**Fig. 1:**
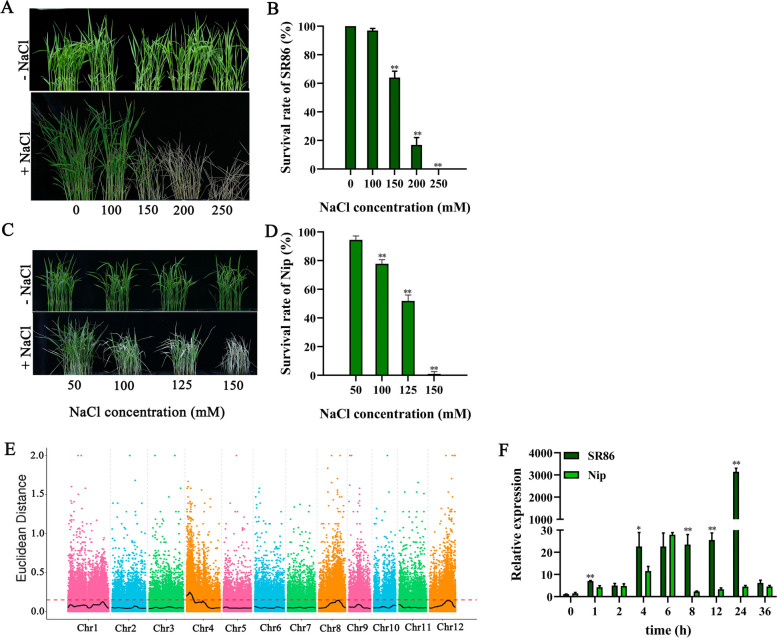
Phenotypic and QTL analysis of SR86 and Nip plants under NaCl stress. **A** Phenotype of SR86 seedling grown under different salt concentrations (0–250 mM). **B** The survival rate of SR86 plants. **C** Phenotype of Nip rice grown under different salt concentrations (50–150 mM). **D** The survival rate of Nip plants. **E** Distribution map of SNP-index values of the mixed pool of F_2_ plants. The SNP-index or ΔSNP-index value is calculated via each point in the figure. The black line indicates the fitted SNP-index or ΔSNP-index values, and the red dashed line is the 99th percentile threshold. **F** Temporal expression pattern of *LOC_Os04g03320* in SR86 and Nip plants under NaCl stress. In (**B**, **D**, **F**), data present mean ± standard deviation of three biological replicates. Asterisks indicate statistically significant differences (*P < 0.05, **P < 0.01; Student’s *t*-test)

To analyze the salt-tolerant phenotypes of SR86 and Nipponbare (Nip), SR86 and Nip rice were treated with different concentrations of salt. In the 150 mM NaCl treatment, SR86 plants showed a survival rate of 70%, while Nip showed no survival (Fig. 1A–D). To mine the salt tolerance-related genes in SR86, 30 extremely salt-tolerant and 30 extremely salt-sensitive plants were selected from the F_2_ progeny of the SR86 × Nip cross, and mixed bulks were generated. BSA-seq analysis revealed salt tolerance related candidate region on chromosome 4, chromosome 8 and chromosome 12 (Fig. 1E). Based on the threshold line, chromosome 4 was judged to have the relevant to the target trait (Fig. 1E). Functional annotation analysis revealed that this region contained a putative salt stress-related gene, *LOC_Os04g03320*. Real-time quantitative PCR (RT-qPCR) analysis suggested that the expression level of *LOC_Os04g03320* was significantly higher in SR86 than that in Nip in the 150 mM NaCl treatment (Fig. 1F). Therefore, we selected *LOC_Os04g03320* as the candidate salt tolerance-related gene. Because the protein encoded by this gene was a JRLs protein, we named this gene as *OsJRL45*.

### *OsJRL45* gene expression analysis and OsJRL45 protein subcellular localization assay

**Fig. 2:**
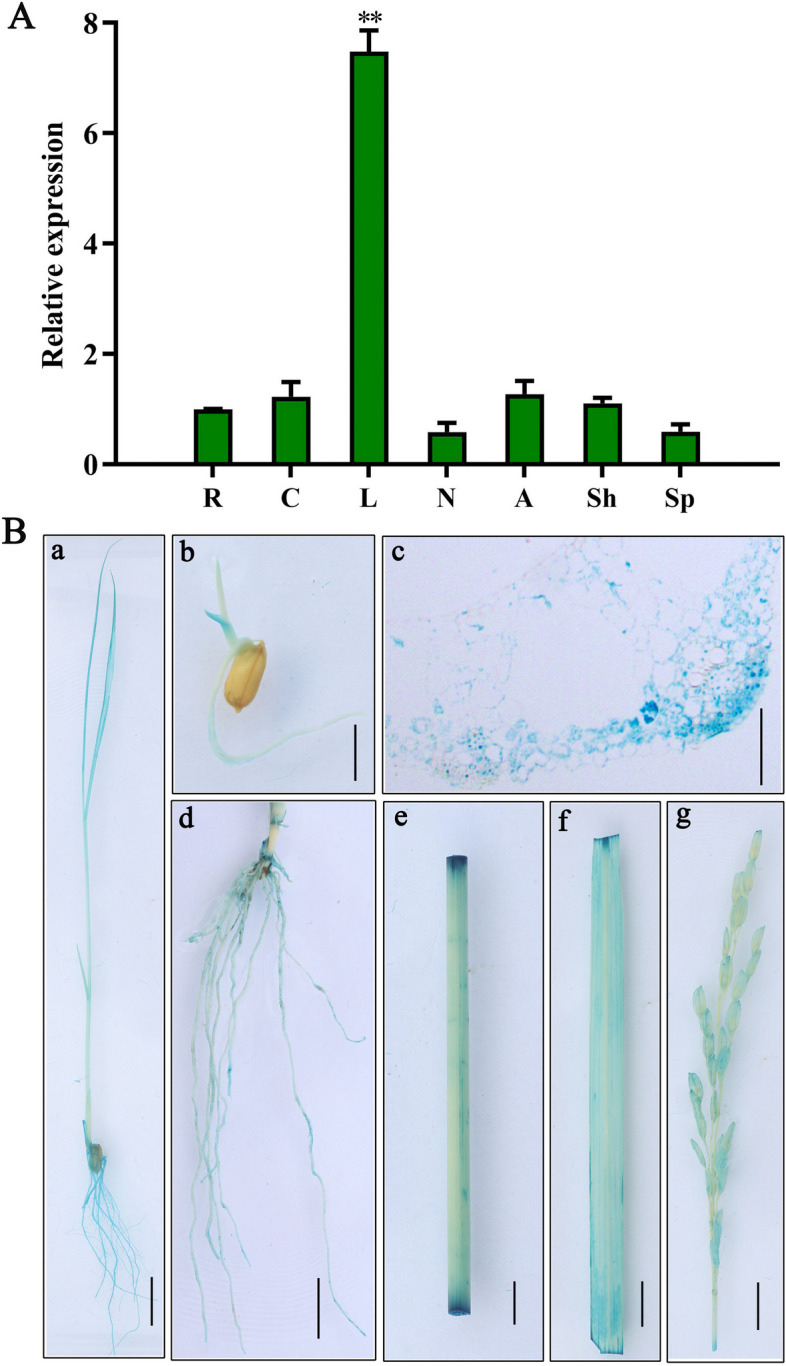
Expression analysis of *OsJRL45*. **A** Expression levels of *OsJRL45* in different tissues of Nip rice including root (R), culm (C), leaf (L), node (N), anther (A), glume (Sh), and young panicle (Sp). **B** GUS staining analysis. GUS staining of seedling (a), bud sheath (b), leaf cross-section (c), root (d), culm (e), leaf (f), and panicle (g) is shown. Bars in a-b, d-g = 1 cm and bar in c = 20 μm

**Fig. 3:**
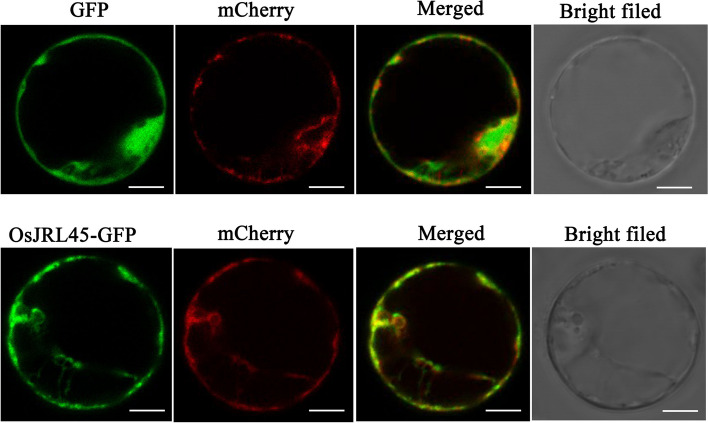
Subcellular localization analysis of *OsJRL45* in Nip protoplasts. Rice protoplasts were transformed with empty vector and the *OsJRL45*‐GFP fusion protein, respectively, whose expression was driven by the 35S promoter. An endoplasmic reticulum localization marker HDEL reported earlier [51, 52] was co‐transfected with the 35S: GFP protein. Protoplasts transformed with GFP alone served as a control. Scale bar = 10 μm

The expression pattern of *OsJRL45* was investigated in different tissues of Nip rice. The results of RT-qPCR results suggested that *OsJRL45* showed the highest expression level in leaves (Fig. 2A). To in-depth clarify the tissue-specific expression pattern of *OsJRL45*, a 2.5 kb fragment of the *OsJRL45* promoter driving the *β-glucuronidase* (GUS) reporter gene was transformed into Nip. GUS staining analysis suggested that this gene was expressed in the bud sheath at the bud stage. The whole plant at the seedling stage and the root, culm, leaf, and panicle at the reproductive growth stage were stained (Fig. 2B). Leaves were stained darker than the other tissues, and the vascular system were also stained. The subcellular localization assay performed using rice protoplasts indicated that the *OsJRL45*-GFP fusion protein was mainly targeted to the endoplasmic reticulum (Fig. 3).

### *OsJRL45* positively regulates salt tolerance at the germination and seedling stages

**Fig. 4:**
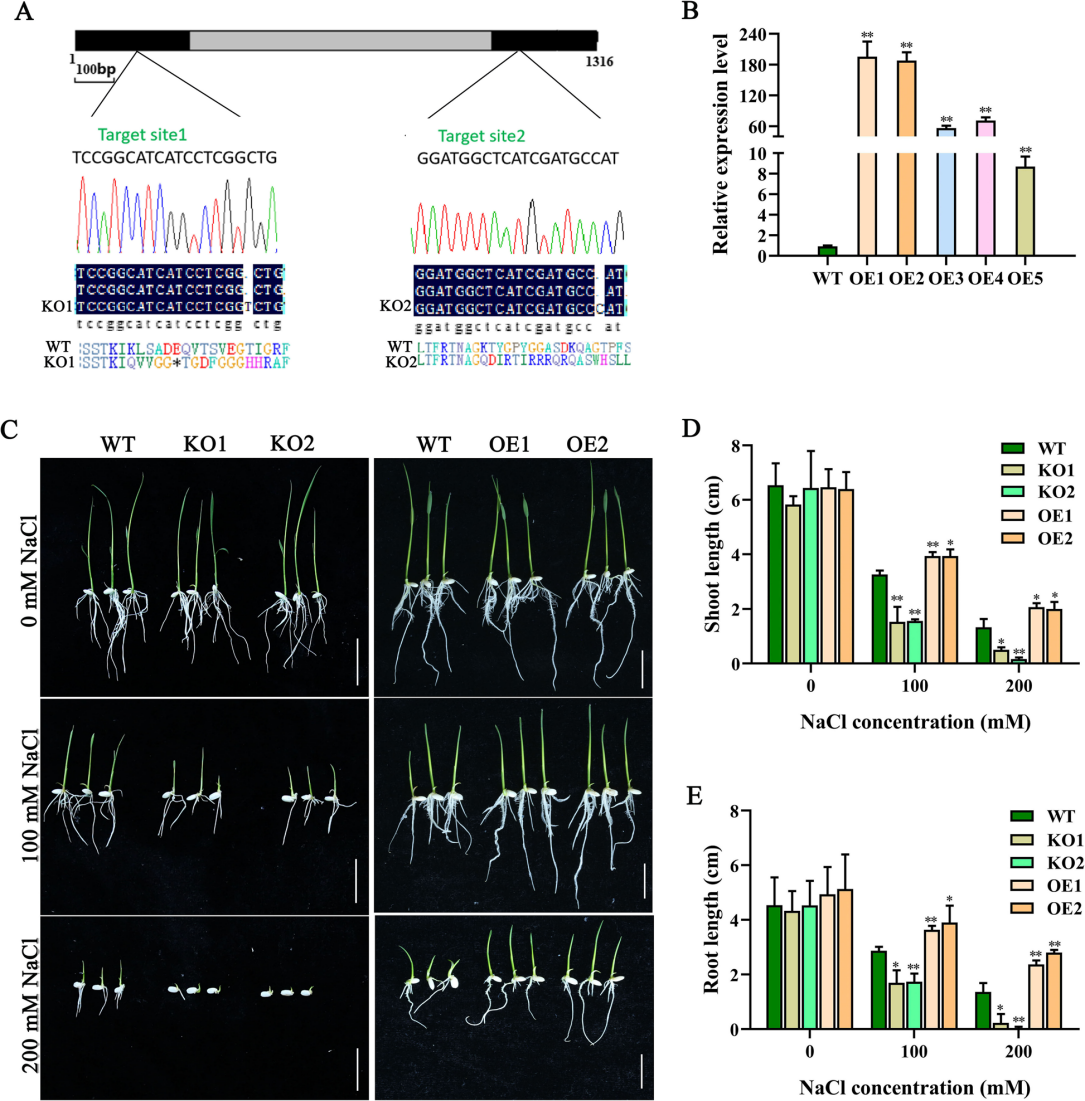
*OsJRL45* promotes seed germination and seedling growth of rice under salt stress. **A** A schematic diagram of CDS coding sequence structure of *OsJRL45*. Black and gray rectangles boxes respectively represent the exons and intron, and the CRISPR/Cas9 targeting site of the *osjrl45* knockout (KO) plants are showed in the figure. The 1 nt insertions in *OsJRL45* exons in two knockout mutants are also indicate. The asterisks are used to show the premature stop codon in the deduced amino acid sequence of the mutant OsJRL45 protein. **B** Transcription levels of OsJRL45 in the leaves of WT and *OsJRL45* overexpression plants (OE1-5). **C**-**D** Germination test (**C**), shoot length (**D**), and root length (**E**) of WT, KO1, KO2, OE1, and OE2 genotypes grown in different concentrations of NaCl. Seeds were dehulled, and incubated first on Murashige and Skoog (MS)-agar medium for 2 d and then on MS-agar medium containing 100 mM or 200 mM salt for 7 d. Data present mean ± SD of three biological replicates. Asterisks show statistically significant differences (*P < 0.05, **P < 0.01; Student’s *t*-test)

To investigate whether *OsJRL45* plays a role in salt tolerance, knockout (KO) mutants of *OsJRL45* were created using CRISPR/Cas9 method in the rice Nip background. The KO1 plant had a 1 nt insertion in the first exon of *OsJRL45*, leading to premature termination of protein translation, while the KO2 plant had a 1 nt insertion in the second exon of *OsJRL45*, causing amino acid shift mutations (Fig. 4A). Five independent *OsJRL45* overexpression plants (OE1–5) also were generated Real-time quantitative PCR showed that *OsJRL45* was significantly upregulated in all five OE plants compared with the wild type (WT; Nip) (Fig. 4B). Finally, two transgenic lines with high *OsJRL45* expression levels, OE1 and OE2, were selected for further analysis.

The ability of seeds to germinate under high salinity conditions is an important indicator of salt tolerance [53]. Germination experiments were performed using the seeds of WT plants, *Osjrl45* mutant lines (KO1 and KO2), and *OsJRL45*-OE lines (OE1 and OE2) under control (0 mM NaCl) and salt treatments (100 and 200 mM NaCl) (Fig. 4C–E). Under normal conditions, the WT, KO1, KO2, OE1, and OE2 genotypes showed no difference in the seed germination rate and seedling growth (Fig. 4C). In the presence of 100 mM NaCl, compared with the WT, the shoot and root lengths of knockout mutants seedlings were significantly reduced, while those of overexpression lines seedlings were significantly increased (Fig. 4C-E). Under 200 mM NaCl stress, the KO1 and KO2 lines showed seed germination but almost no seedling growth (Fig. 4C-E).

**Fig. 5:**
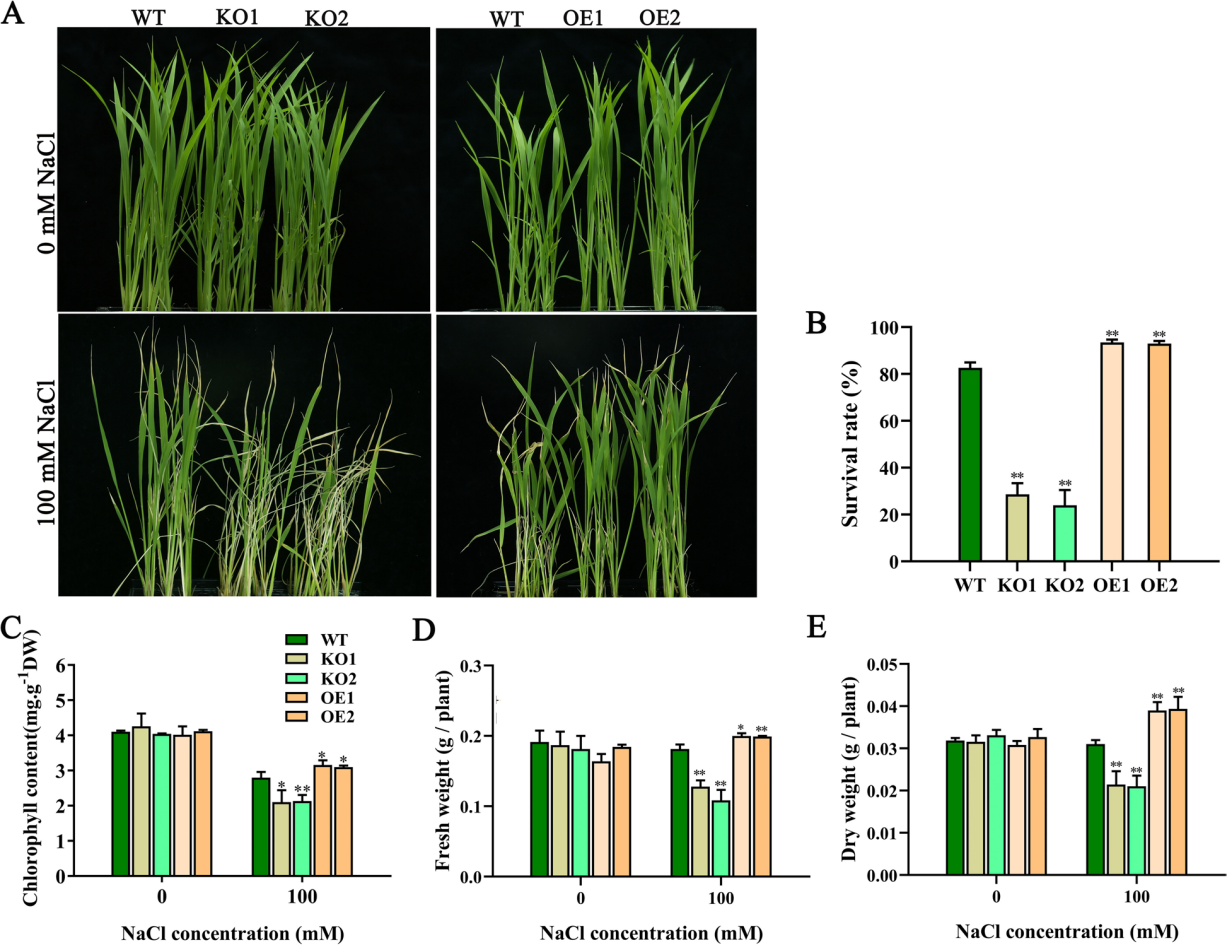
*OsJRL45* increases salt tolerance in rice at the seedling stage. **A**-**E** Phenotype under the NaCl treatment condition (**A**), survival rate (**B**), chlorophyll content (**C**), fresh weight (**D**), and dry weight (**E**) of WT and transgenic rice seedlings grow under 0 mM salt and 100 mM salt treatments. In (**B**-**E**), data represent the mean ± SD of three independent replicates, and asterisks show statistically significant differences compared with the WT (*P < 0.05, **P < 0.01)

Next, we analyzed the salt tolerance of WT and all knockout and overexpressed transgenic rice at the seedling period. Under normal conditions, the WT, KO1, KO2, OE1, and OE2 genotypes showed no difference (Fig. 5A). Under salt stress, the knockout plants indicated severe wilting, while the overexpressed plants grew better than WT plants (Fig. 5A). The chlorophyll content, fresh weights and dry weights of KO1 and KO2 plants were significantly lower after treatment than before treatment, while the chlorophyll contents of OE1 and OE2 plants were lower and their fresh weights and dry weights were significantly higher (Fig. 5C-E). The survival rates (Fig. 5B), chlorophyll contents (Fig. 5C), fresh weights (Fig. 5D), and dry weights (Fig. 5E) of knockout plants seedlings were significantly lower than those of the WT plants, while those of overexpressed transgenic rice seedlings were higher under salinity stress treatments. Collectively, these results suggest that OsJRL45 positively regulates salt tolerance at the germination and seedling stages.

### *OsJRL45* improves salt tolerance in rice at the reproductive stage

**Fig. 6:**
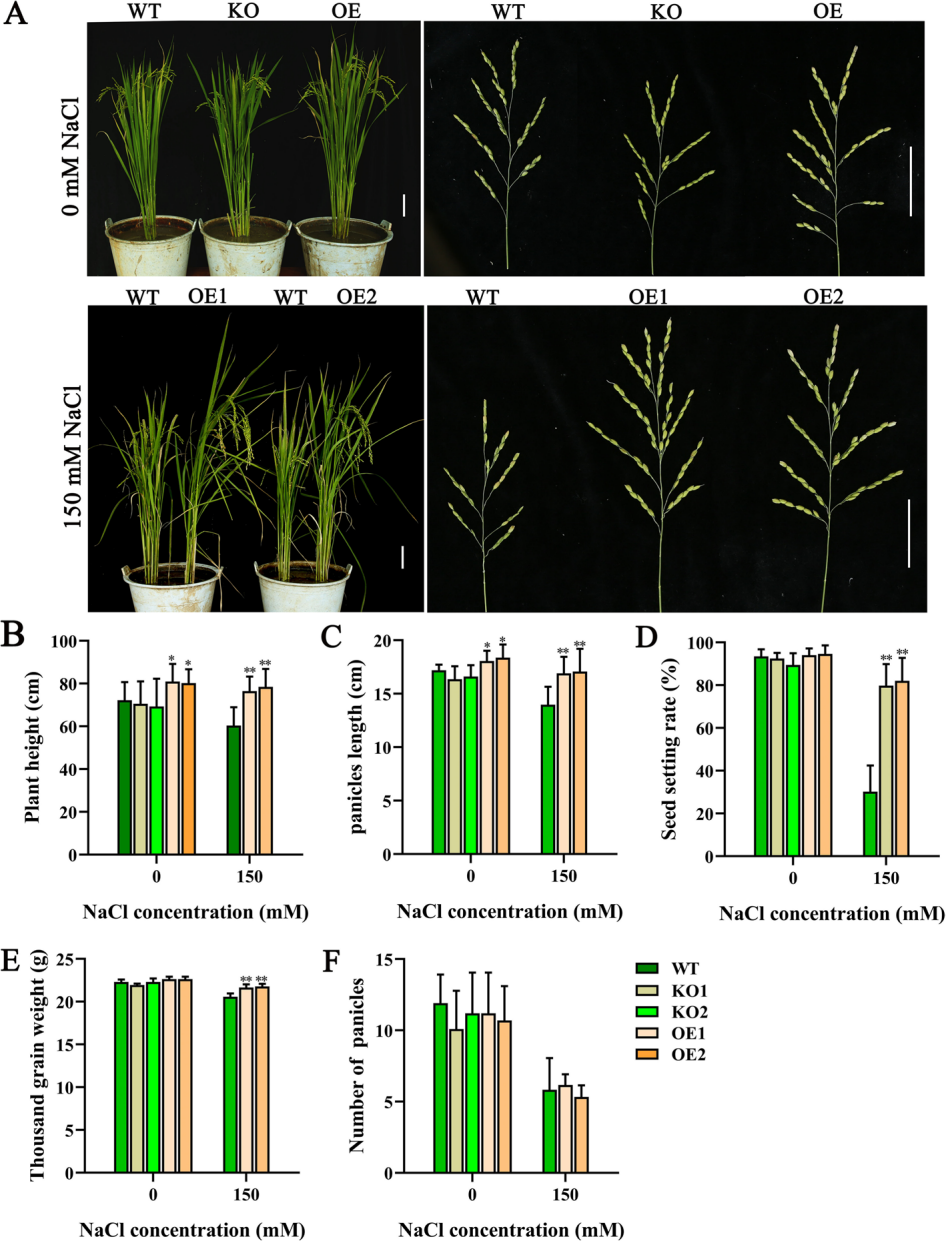
*OsJRL45* enhances salt tolerance of rice during the reproductive period. **A**-**F** Phenotype (**A**), plant height (**B**), main panicle length (**C**), seed setting rate (**D**), thousand-grain weight (**E**), and effective panicle number (**F**) of WT and transgenic plants grow under 0 mM salt and 100 mM salt treatment conditions. In A, scale = 5 cm. In B-F, results present mean ± SD of ten biological replicates. Asterisks show statistically significant differences (*P < 0.05, **P < 0.01; Student’s *t*-test)

To verify whether *OsJRL45* affects salt tolerance during reproductive period, the WT and KO plants phenotypes and agronomic traits were measured under 0 mM salt and 150 mM salt treatments (Fig. 6). Under normal conditions, the plant height and panicle length of overexpressed transgenic lines were greater than those of the WT rice; however, no significant difference was found between the KO and WT plants (Fig. 6A, B, C). Under salinity treatment condition, all knockout mutant plants died, while the overexpression plants suggested significantly greater panicle length, seed setting rate, and 1000-grain weight than the WT plants (Fig. 6C-E). The panicle number was not significantly different between WT, KO and OE plants (Fig. 6F). These results indicate that *OsJRL45* overexpression enhances the salt tolerance of rice at the reproductive period and increases rice yield under salt stress.

### *OsJRL45* enhances salt tolerance of a salt-sensitive *indica* cultivar

**Fig. 7:**
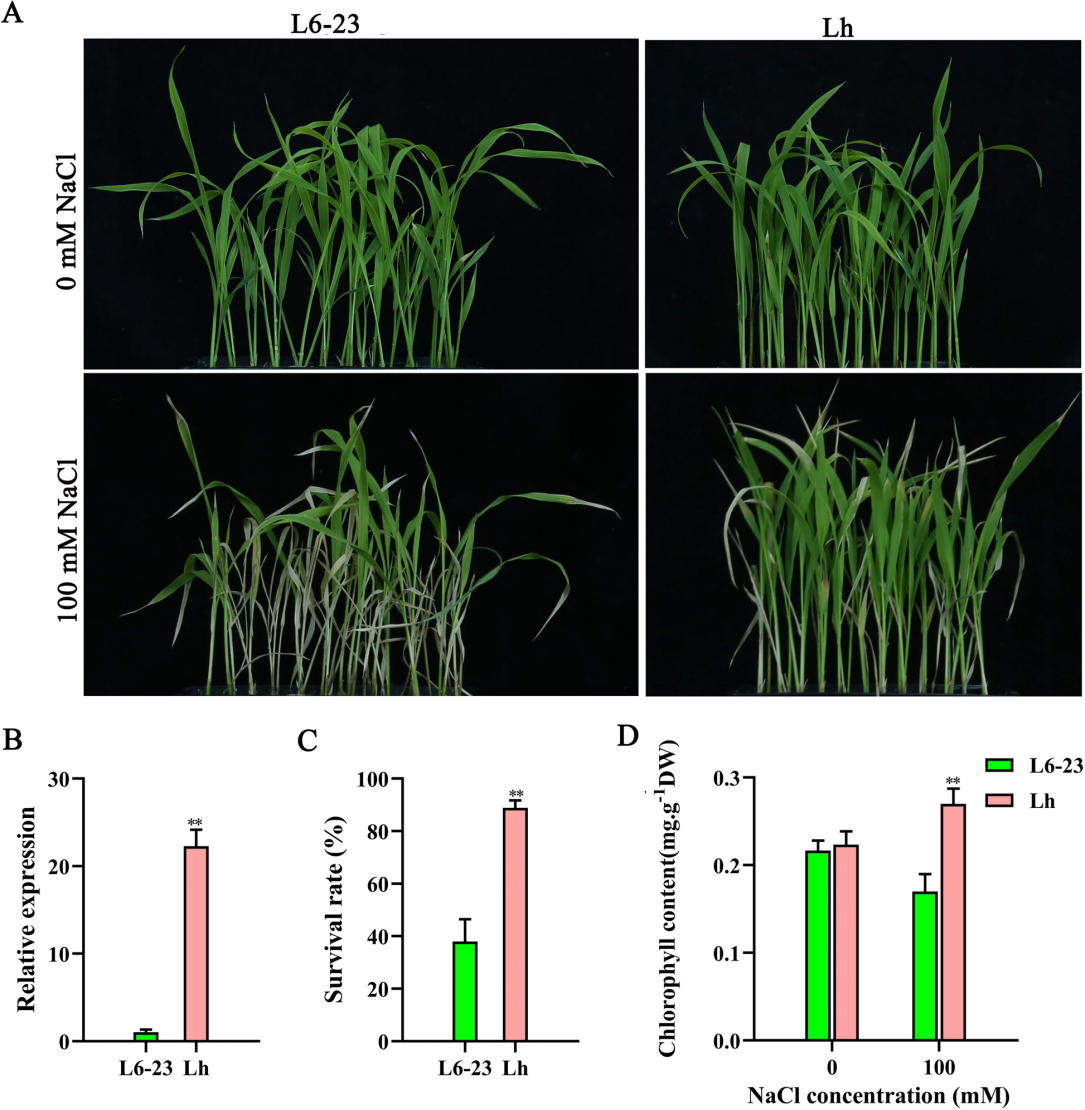
*OsJRL45* improves the salt tolerance of the salt-sensitive *indica* rice cultivar L6-23 at the seedling stage. Wild-type (L6-23) and *OsJRL45*-overexpressing L6-23 (Lh) seedlings were exposed to 100 mM salt for 3 d. **A** Phenotypic evaluation of L6-23 and Lh plants before and after salt treatment. **B** Expression analysis of *OsJRL45* in L6-23 and Lh plants. **C** Survival rate of L6-23 and Lh plants under NaCl treatment. **D** Chlorophyll content of L6-23 and Lh plants under 0 mM salt and 100 mM salt treatments. In (**B**-**D**), data indicate mean ± SD. Asterisks represent statistically significant differences (*P < 0.05, **P < 0.01)

To elucidate its biological function, the *OsJRL45* gene isolated from SR86 was transformed into the salt-sensitive *indica* cultivar L6-23. The promoter of *OsJRL45* was cloned into the pCAMBIA1300 vector, and the resultant construct was transformed into L6-23 via *Agrobacterium*-mediated transformation to create transgenic rice (named Lh). The L6-23 plants suggested severe wilting, while the Lh plants grew better than the L6-23 rice under salt stress (Fig. 7A). RT-qPCR results indicated that the expression level of *OsJRL45* was significantly higher in Lh transgenic rice than in L6-23 plants (Fig. 7B). Compared with the L6-23 plants, the survival rate (Fig. 7C) and chlorophyll content (Fig. 7D) of Lh plants were significantly higher under salt stress conditions.

**Fig. 8:**
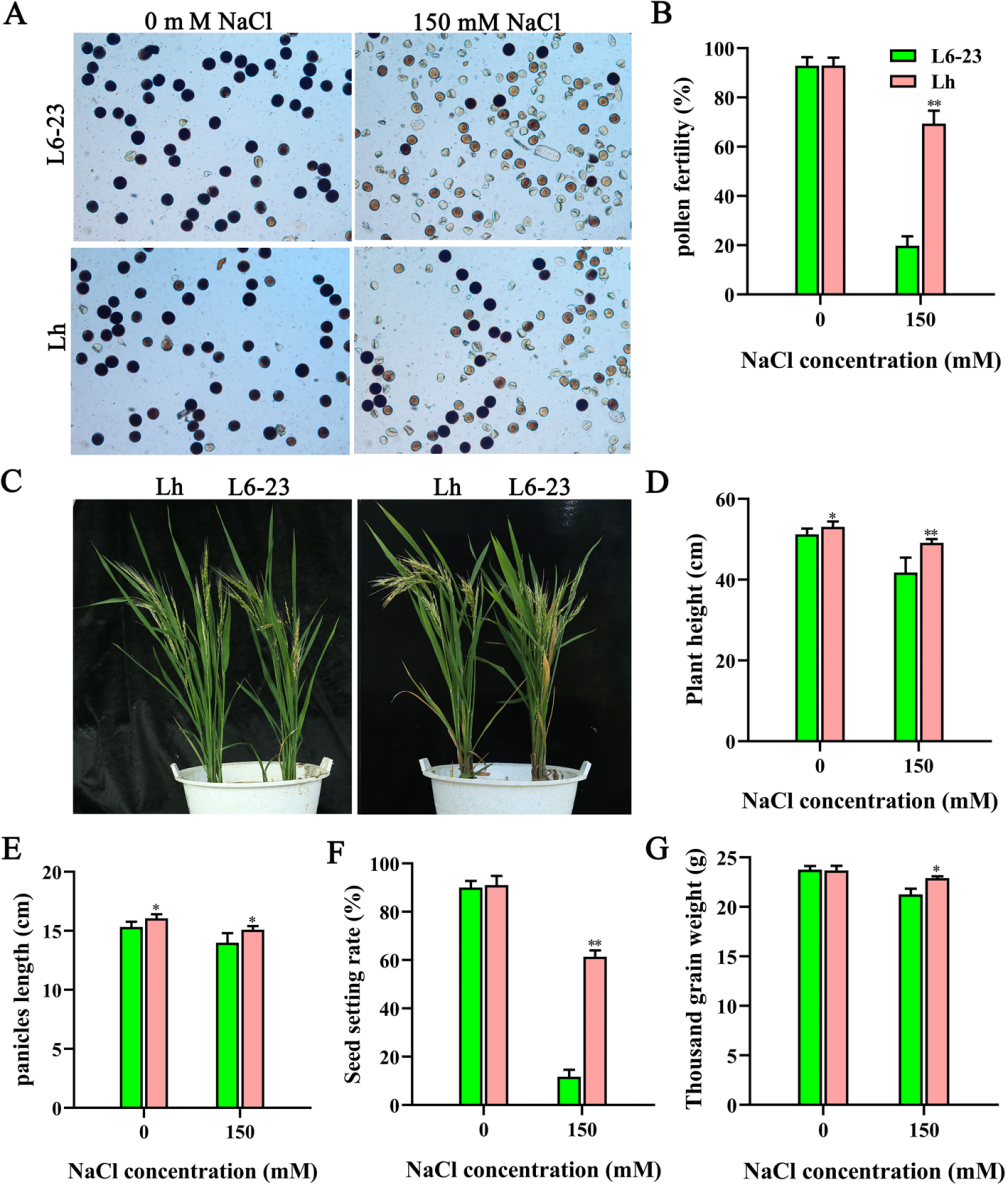
*OsJRL45* enhances the salt tolerance of the salt-sensitive *indica* rice cultivar L6-23 during the reproductive period. rice at the tillering period were transplanted into soil containing no salt or 150 mM NaCl. **A** Pollen phenotype under no salt or 150 mM NaCl conditions. **B** Pollen fertility rate no salt or 150 mM NaCl conditions. **C** Phenotype of L6-23 and Lh plants at maturity under normal and salt stress conditions. **D** Plant height. **E** Panicle length. **F** Seed setting rate. **G** 1000-grain weight. In (**B**, **D**-**G**), data indicate mean ± SD. Asterisks represent statistically significant differences (*P < 0.05, **P < 0.01)

Next, we analyzed the salt tolerance of L6-23 and Lh transgenic rice at the reproductive stage under normal and salt stress (150 mM salt) conditions (Fig. 8). The pollen fertility of Lh plants was significantly greater than that of L6-23 plants under salt stress conditions (Fig. 8A, B). Compared with the L6-23 rice, the plant height (Fig. 8C, D) and panicle length (Fig. 8E) of Lh plants were significantly greater under both normal and salt treatment conditions, while the seed setting rate (Fig. 8F) and 1000-grain weight (Fig. 8G) of Lh transgenic rice were significantly greater only under salt treatments; notably, the seed setting rate of Lh plants was increased by 60% compared with the L6-23 plants (Fig. 8F). Collectively, these results show that *OsJRL45* can significant enhance the salt tolerance.

### *OsJRL45* enhances the antioxidant capacity of rice under salt stress

**Fig. 9:**
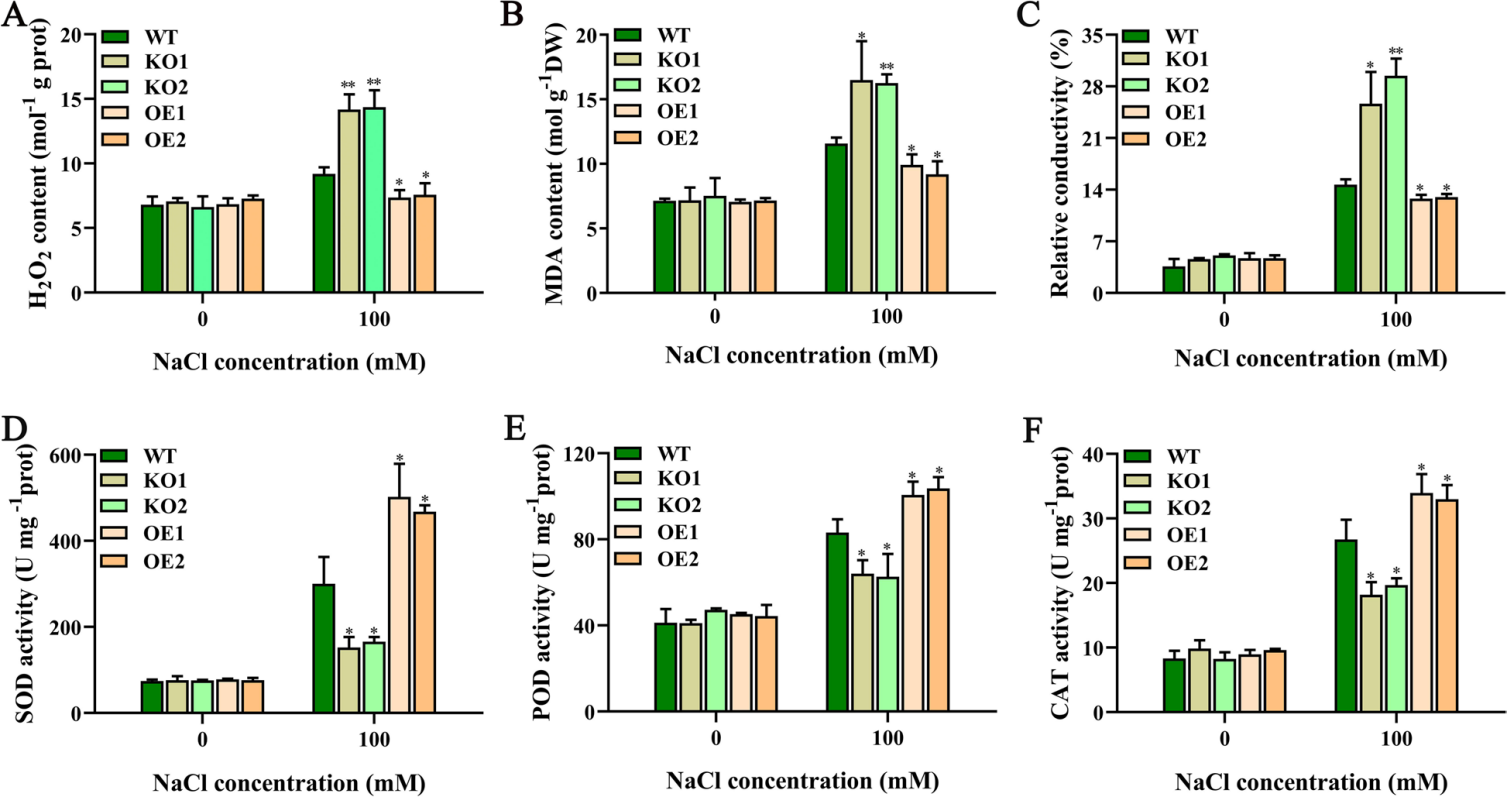
*OsJRL45* increases the antioxidant capacity of rice under NaCl treatment. Fourteen-day-old WT, KO1, KO2, OE1, and OE2 plants were treated with 0 mM or 100 mM salt for 2 days before measuring various physiological and biochemical indicators. **A** H_2_O_2_ content. **B** MDA content. **C** Relative electrical conductivity. **D** SOD activity. **E** POD activity. **F** CAT activity. Data indicate mean ± SD. Asterisks represent statistically significant differences (*P < 0.05, **P < 0.01)

To further elucidate the molecular function of plant response to NaCl stress, we measured various physiological and biochemical indexes of wild-type and transgenic rice under normal and salt treatment conditions. No significant differences were suggested between WT, KO, and OE plants under normal conditions (Fig. 9). Under salt stress conditions, compared with WT plants, the KO mutant plants suggested significantly higher hydrogen peroxide (H_2_O_2_) content, malondialdehyde (MDA) content and electrical conductivity (Fig. 9A-C) but significantly lower superoxide dismutase (SOD), peroxidase (POD), and catalase (CAT) activities in Fig. 9D-F; the trends observed in OE lines were the opposite of those observed in KO plants. Collectively, these date show that *OsJRL45* may be involved in suppressing damage caused by oxidative stress, thereby enhancing the salt tolerance of rice.

### *OsJRL45* regulates Na^+^-K^+^ homeostasis under salt stress

**Fig. 10:**
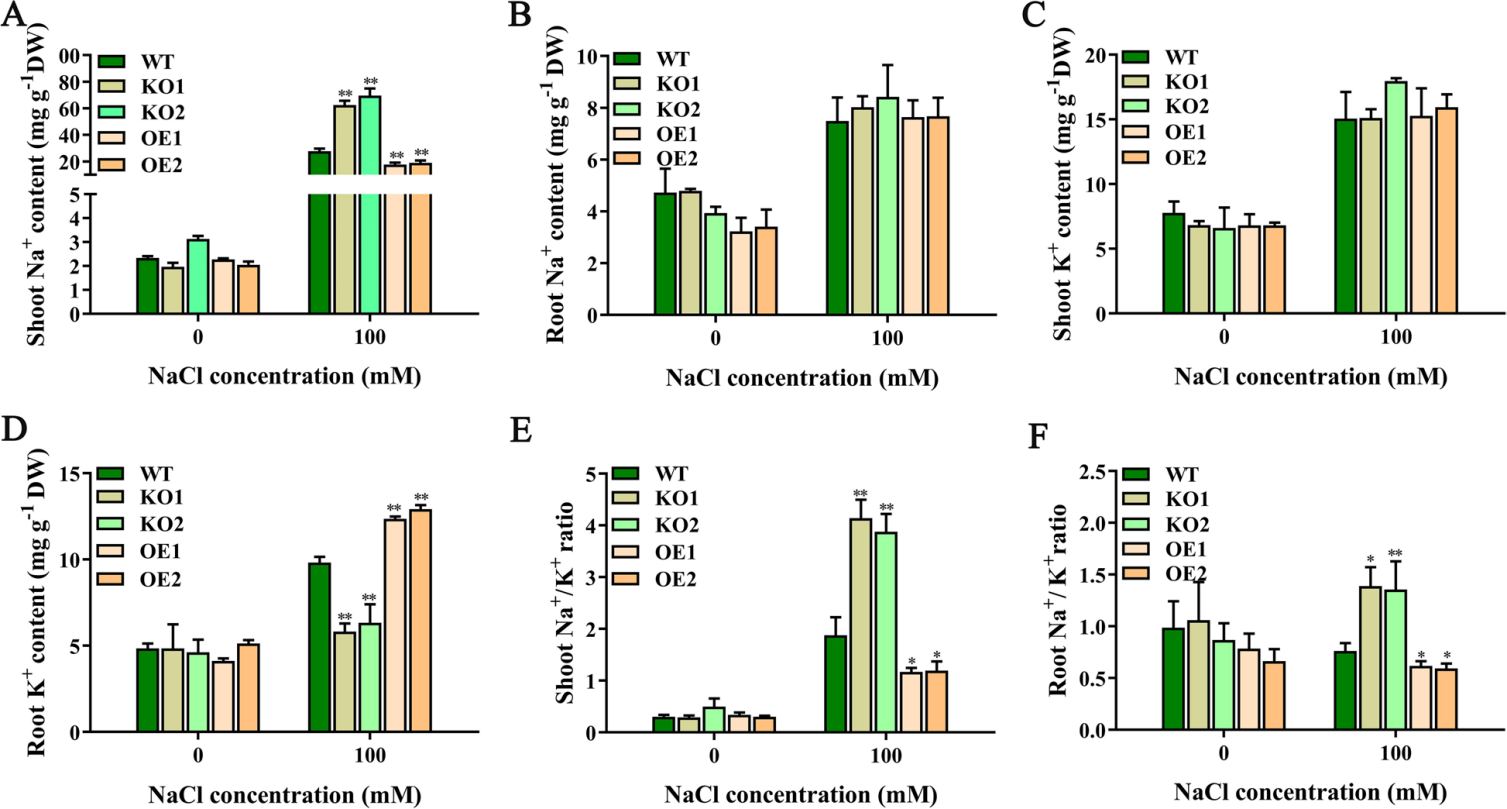
*OsJRL45* affects Na^+^-K^+^ homeostasis. WT, knockout and overexpression of transgenic plants were treated with 100 mM salt in hydroponic medium for 2 days. **A** Na^+^ content in shoots. **B** Na^+^ content in roots. **C** K^+^ content in shoots. **D** K^+^ content in roots. **E** Na^+^/K^+^ ratio in shoots. **F** Na^+^/K^+^ ratio in roots. DW, dry weight. Results indicate mean ± SD. Asterisks represent statistically significant differences (*P < 0.05, **P < 0.01)

To determine whether *OsJRL45* is involved in Na^+^-K^+^ homeostasis under salt treatment conditions, we measured the contents of Na^+^ and K^+^ in the shoots and roots of WT and transgenic rice plants treated with no salt (0 mM NaCl) and 100 mM NaCl (Fig. 10). No significant differences were found in the Na^+^ and K^+^ contents of the shoots and roots of WT and transgenic rice plants under normal conditions (Fig. 10). However, under salinity treatment, compared with WT plants, the KO lines indicated significantly higher content of Na^+^ in shoots and significantly lower content of K^+^ in roots, while the OE lines suggested the opposite results (Fig. 10A, D). Consequently, in both shoot and root tissues, the Na^+^/K^+^ ratio was significantly higher in KO knockoutmutants and significantly lower in OE lines compared with the WT under salt stress conditions (Fig. 10E, F). These results show that *OsJRL45* was involved in regulating Na^+^/K^+^ homeostasis to enhance salt tolerance of rice.

### Identification of genes and interaction networks regulated by *OsJRL45*

**Fig. 11:**
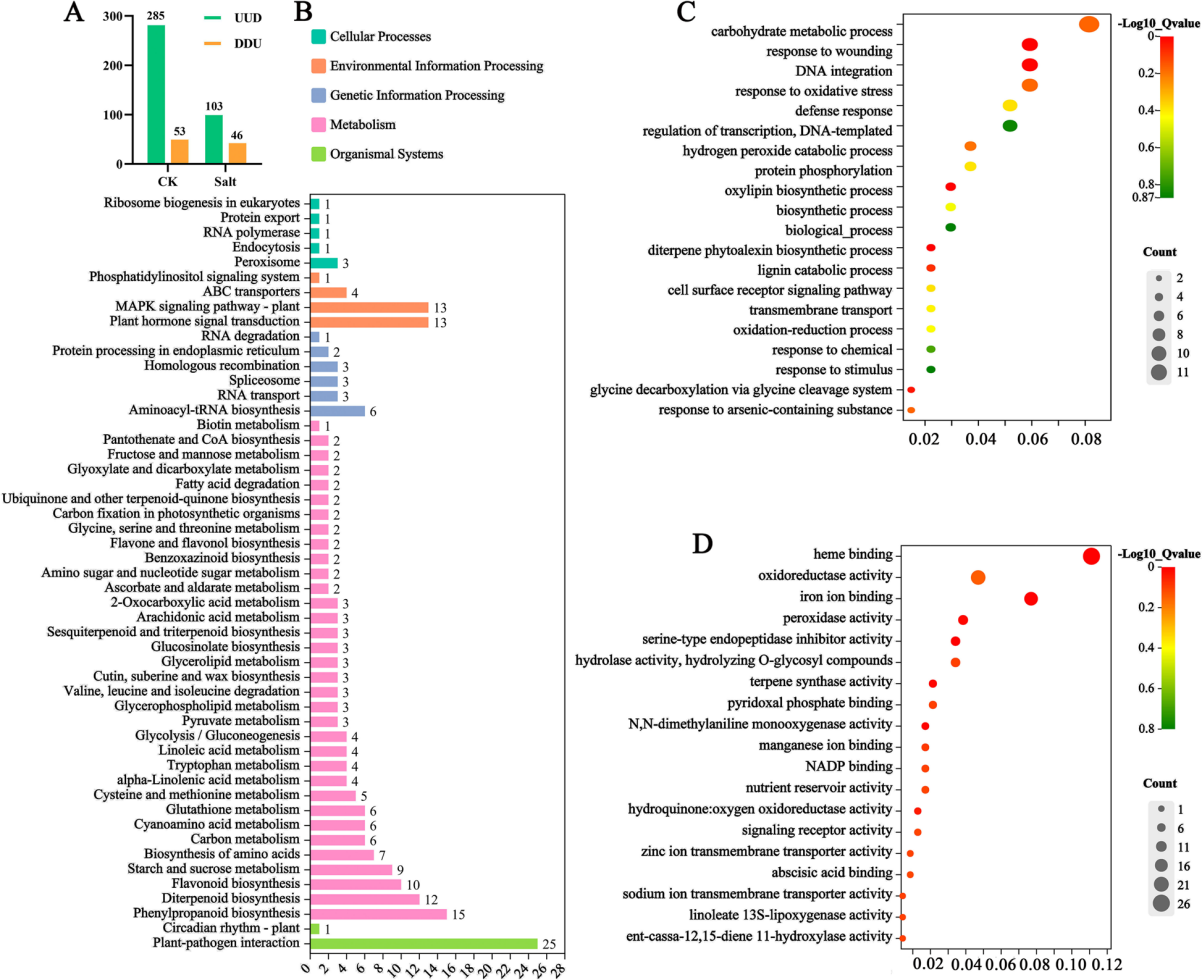
GO and KEGG pathway analyses. **A** Statistics of the number of DEGs identified under control (CK; 0 mM salt) and salt stress (salt; 100 mM salt) treatments. ‘UUD’ represents up-regulated DEGs; ‘DDU’ represents down-regulated DEGs. **B**-**D**) Results of KEGG (**B**) and GO (**C**, **D**) enrichment analyses of DEGs [54–56]. The GO terms enriched in the biological process category (**C**) and molecular function category (**D**) are shown

To analyze the molecular mechanisms of *OsJRL45*, we conducted transcriptome analysis of WT and KO rice plants grown under 0 mM salt and 100 mM salt treatments. Compared with the WT, 285 genes were upregulated and 53 were downregulated in KO lines under normal conditions, and 103 genes were upregulated and 46 were downregulated in KO plants under salt stress conditions (Fig. 11A). Next, we conducted functional enrichment analysis of the differentially expressed genes (DEGs) between WT and KO plants under the two conditions using the Kyoto Encyclopedia of Genes and Genomes (KEGG) and Gene Ontology (GO) databases. The results of KEGG pathway analysis results suggested that most of DEGs were enriched in plant-pathogen interaction, hormone signal transduction, MAPK signaling pathway, and the biosynthesis of phenylpropanoids, diterpenoids, and flavonoids (Fig. 11B). The results of GO analysis results revealed that these DEGs were associated with oxidative stress, oxidoreductase activity, and ion transport activities (Fig. 11C, D).

**Fig. 12:**
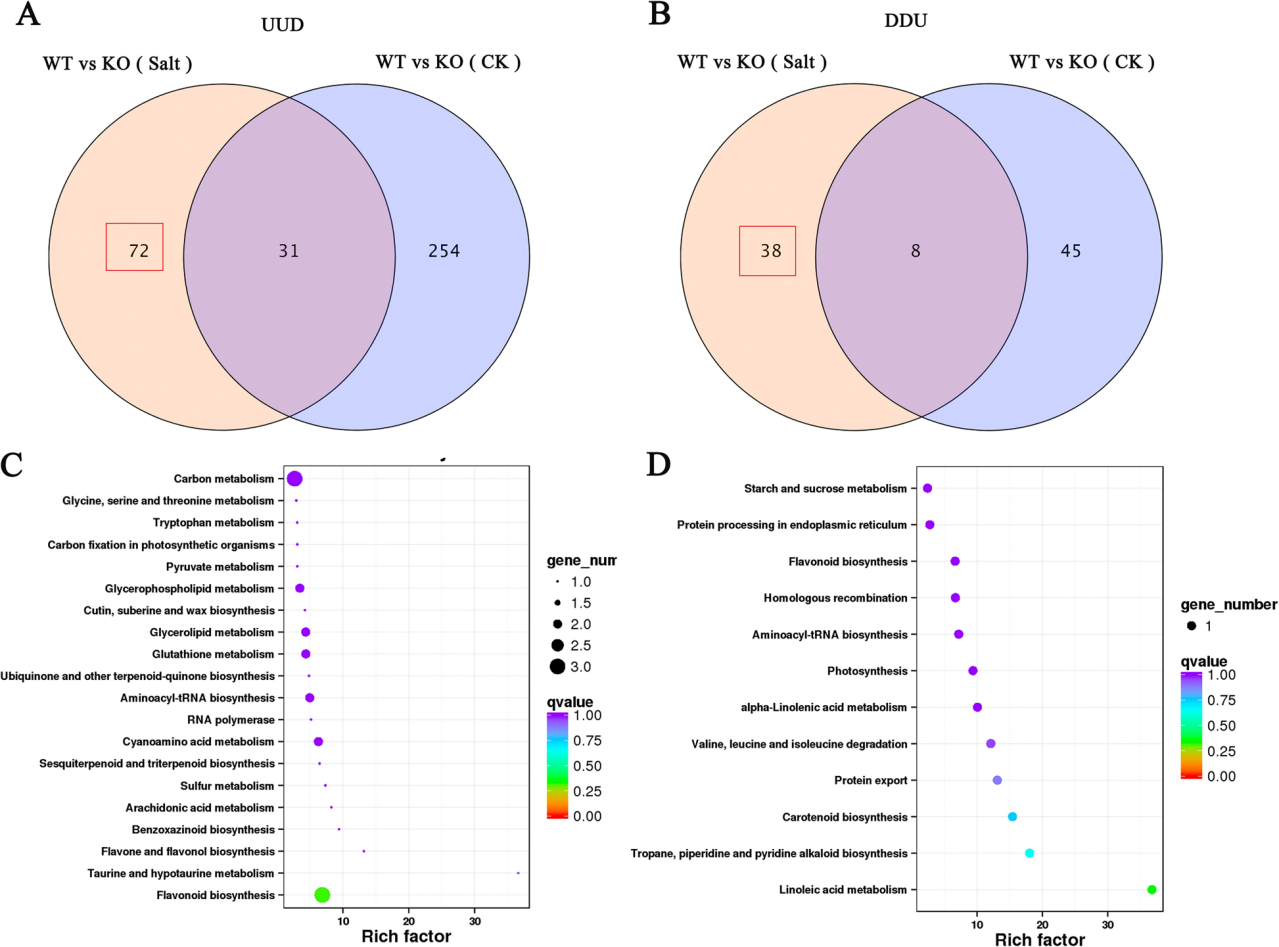
Transcriptomic analysis of DEGs regulated by *OsJRL45*. **A** Venn diagram exhibiting the number of DEGs upregulated (fold change > 2) in KO mutants under control (CK; 0 mM salt) and salt treatment (Salt; 100 mM salt) conditions. **B** Venn diagram exhibiting the number of DEGs downregulated (fold change > 2) in KO mutants under control (CK; 0 mM salt) and salt treatment (Salt; 100 mM salt) conditions. **C** KEGG enrichment diagram of DEGs upregulated after salt treatment. **D** KEGG enrichment diagram of DEGs downregulated after the salt treatment. KEGG imagery is available in Kanehisa Laboratories [54–56]. Three biological replicates (*n* = 3) were performed for each replicate

Further analysis revealed that 72 genes were upregulated in the KO mutants only after the salt treatment (UUD, Fig. 12A, Supplementary Data 2). Similarly, 38 genes were downregulated in the KO mutants only after the salt treatment (DDU, fold change ≥ 2) (Fig. 12B, Supplementary Data 3). Thus, 110 DEGs were directly related to salt stress. KEGG pathway enrichment showed that these 110 DEGs were mainly involved in flavonoid biosynthesis, and in linoleic acid and amino acid biosynthesis and metabolism (Fig. 12C, D).

**Fig. 13:**
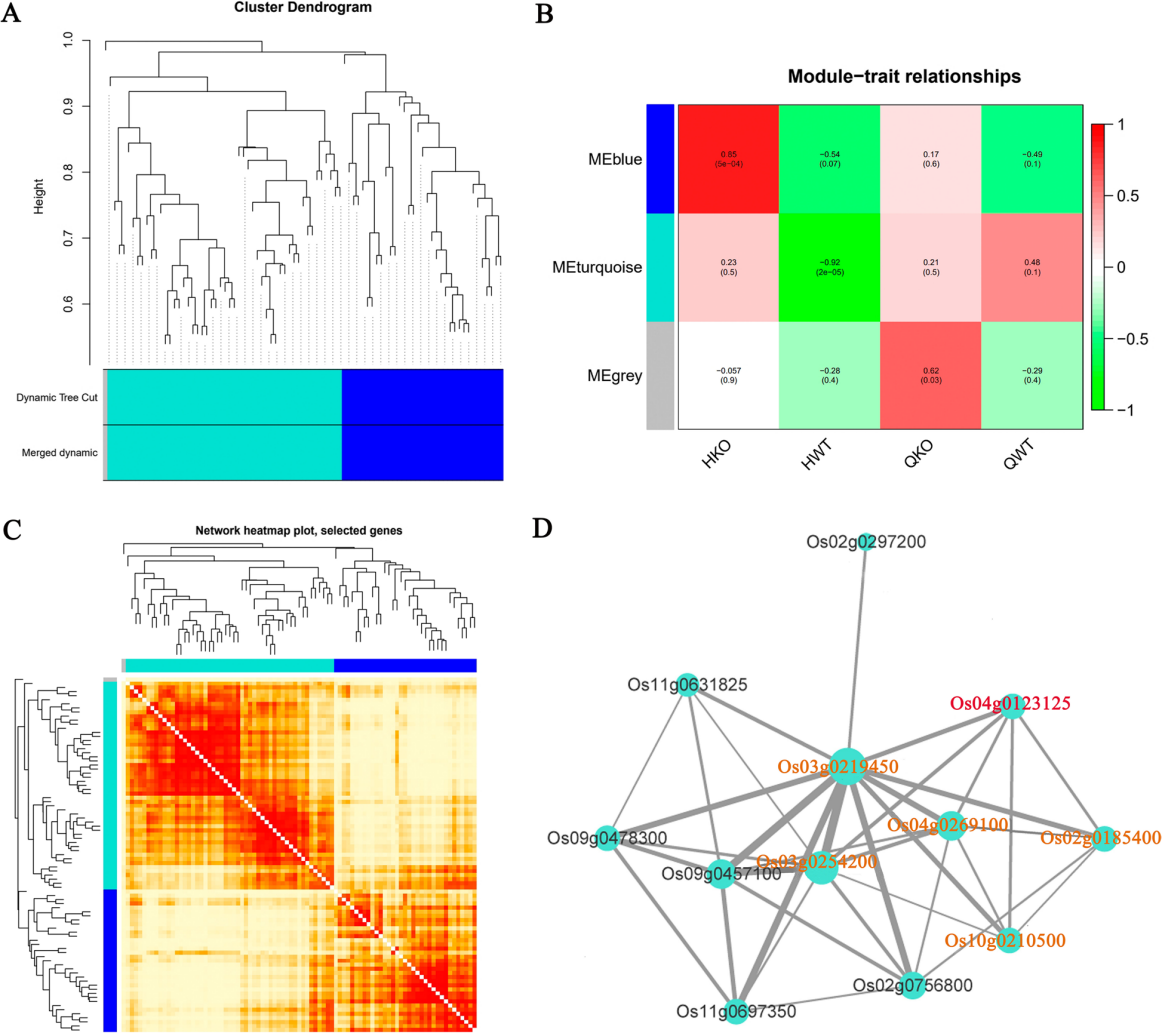
Co-expression network analysis of *OsJRL45*. **A** Clustering and expression map of genes in the WGCNA module. **B** Gene and phenotype data association analysis diagram within the module. “HKO and HWT” represent knockout and WT plants after salt treatment; “QKO and QWT” represent the knockout and WT plants before treatment. **C** Visualized gene network diagram. **D** Co-expression network of *OsJRL45*. Red font indicates the *OsJRL45* gene. Orange fonts indicates the genes that directly associate with *OsJRL45*

**Fig. 14:**
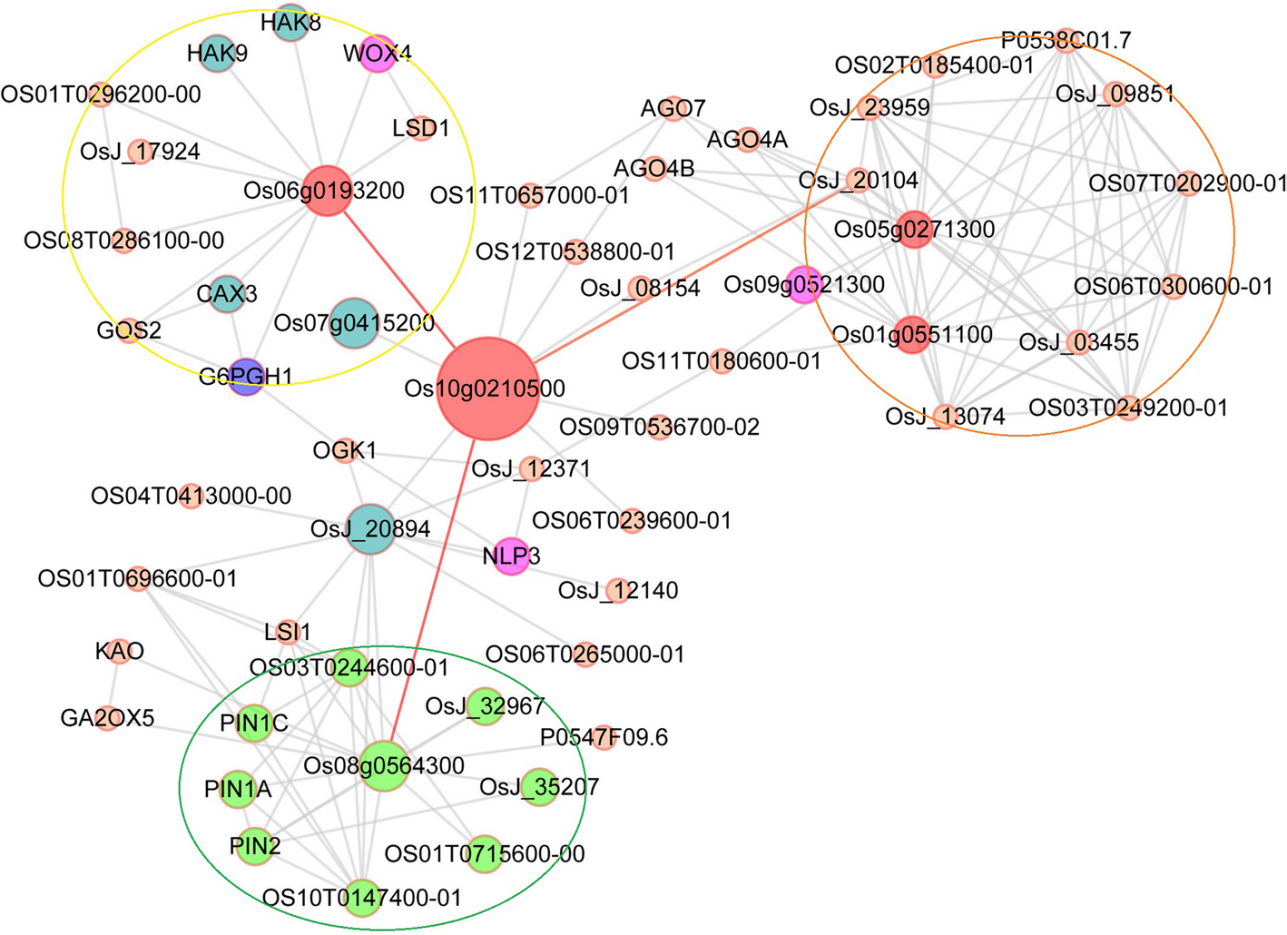
Protein interaction network created using the String website. Round shapes represent proteins (node), red round shapes represent associated proteins and proteins that interact directly with associated proteins; blue round shapes indicate ion transporters; purple round shapes indicate transcription factors; and the line (edge) links two interacting proteins. The network is divided into three modules. Yellow circle indicates proteins mainly involved in stress response and ion transport. Orange circle indicates proteins mainly involved in substance anabolism. Green circle indicates proteins primarily associated with hormone signalling

Co-expression network analysis of these DEGs was performed using the WGCNA and Cytoscape platforms. The results showed that these DEGs were significantly associated with salt stress (Fig. 13A-C). The interaction network revealed five genes related to *OsJRL45* (*Os04g0123125*): *Os02g0185400* (cytochrome P450), *Os03g0219450* (unannotated), *Os03g0254200* (amino acid transporter), *Os04g0269100* (tropinone reductase homolog At2g29360), and *Os10g0210500* (EmA-like transporter) (Fig. 13D). To further explore the functions and relevant pathways of these genes, we created a protein interaction network using the String website (Fig. 14). This network contained stress-responsive proteins, ion transporters, and enzymes (oxidases, peroxidases, and cofactors), with a transmembrane EmA-like transporter, Os10g021050, at the center. In this network, the Os06g0193200 is also annotated as a “putative pectin esterase”, some researches have showed that pectin esterase may play an important role in plant salt tolerance [57–59]. Os06g0193200, which regulates some ion transporters and stress-responsive proteins, showed interaction with Os10g0210500. For example, OsHAK8 is involved in K^+^ uptake and translocation [60], and responds to salt stress [61]; the cax3 mutant is sensitive to salt stress [62]; and G6PGH1 is involved in the response to various abiotic stresses such as high salinity. Additionally, Os08g0564300 which affects the functions of genes related to plant hormone signal transduction showed interaction with Os10g0210500. For example, PIN family proteins are involved in abiotic stress responses and plant hormone signaling [63]. Three transcription factors including WOX4, NLP3, and Os09g0521300 were also identified in the network. Among these, Os09g0521300 and WOX4 have previously been indicated to play important roles in the response to salt and drought stresses and plant hormones [64, 65]. OsJ_20104 is a translation factor GUF1 homolog, and most proteins in this regulatory network are involved in substance synthesis and metabolism. These results suggest that *OsJRL45* may affect the salt tolerance of rice by regulating ion transport- and hormone response-related genes as well as salt stress-responsive genes.

### RT-qPCR expression validation of genes co-expressed with *OsJRL45* under salt stress

**Fig. 15:**
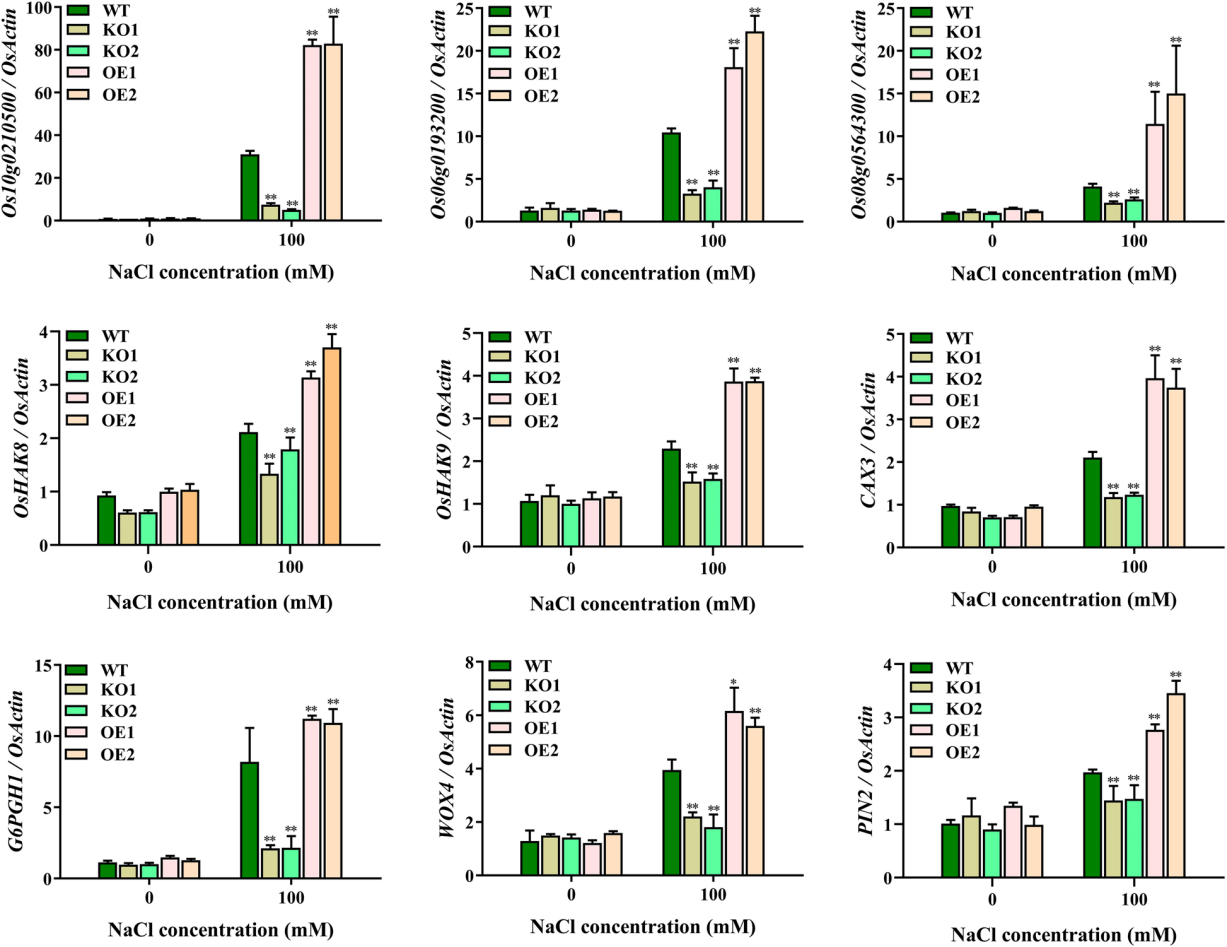
Expression validation of genes co-expressed with *OsJRL45* in WT and knockout rice under 0 mM salt and 100 mM salt treatments by RT-qPCR. The WT and knockout rice plants were treated for 6 h under 100 mM salt conditions. The abscissa indicates the NaCl content, and the ordinate indicates transcription levels, which was calculated with reference to the preceding 2^−ΔΔCT^ method [66]. Data present mean ± SD of three biological replicates. Asterisks show statistically significant differences (*P < 0.05, **P < 0.01; Student’s *t*-test)

To confirm the results of interaction analysis, we verified the expression level of nine genes co-expressed with *OsJRL45* by RT-qPCR. The results showed that all nine genes were downregulated in KO mutant plants and upregulated in OE plants (Fig. 15), as expected.

## Discussion

Salt stress affects rice plants at various growth stages by causing a series of adverse reactions, thus decreasing photosynthesis [67–69], which reduces plant height, tiller number, panicle number, seed setting rate, thousand-grain weight, and ultimately grain yield [70–73]. Among the effects on these agronomic traits, the reduction in the seed setting rate is the most important reason for the reduction in rice yield on saline-alkaline land [74]. Researchers aim to isolate and utilize valuable salt tolerance-related genes for rice cultivar improvement and yield maintenance under salt stress conditions. The role of genes in enhancing the salt tolerance of rice has been shown in many studies, but most of these studies were conducted on rice seedlings [33, 34, 75], HAK family genes were also reported to play an important role in salt tolerance in the reproductive growth stage [28], but the effect of most genes on the salt tolerance of rice plants during reproductive period remains unknown. In this study, we found that *osjrl45* KO mutants were salt-sensitive, and overexpression of *OsJRL45* enhanced salt tolerance (Figs. 4 and 5). Furthermore, the overexpression lines exhibited obvious salt tolerance during reproductive period, because the evidence came from increased panicle length, thousand-grain weight, seed setting rate, and consequently yield under salt stress (Fig. 6). This suggests that *OsJRL45* affected the salt tolerance of rice at all growth stages. This research further enhances our understanding of the salt tolerance mechanism of rice, and could be used as a reference for the creation of high-yielding and salt-tolerant rice varieties.

The role of *JRL* family genes in salt stress tolerance has been found in many different plant species in recent years [45–47]. For example, *OsJRL* upregulates the expression of Na^+^ transporter protein-encoding *HKT* genes under salt stress, which affects the salt tolerance of rice [49]. *Populus euphratica PeJRL* upregulates Na^+^ transporter genes *AHA* and *SOS1* under salt stress conditions, and reduces Na^+^ accumulation, thereby enhancing *P. euphratica* salt tolerance [46]. In the current study, KO mutations of *OsJRL45* increased the Na^+^ content of shoots and decreased the K^+^ content of roots under salt stress conditions; *OsJRL45* overexpression led to opposite results (Fig. 10). The RT-qPCR indicated that the expression level of *OsHAK8* and *OsHAK9* was altered in KO and OE plants (Fig. 15). It has been reported that *HAK* family genes play a key role in mediating K^+^ uptake and translocation, and in regulating salt tolerance [60, 76]. This indicates that *OsJRL45* may affect the uptake and transport of K^+^ under salt stress, thus disturbing the Na^+^-K^+^ homeostasis. We speculate that *OsJRL45* affects the expression of *HAK* family genes under salt stress conditions and regulates K^+^ uptake, thereby achieving Na^+^-K^+^ homeostasis.

Additionally, our results show that *OsJRL45* improves the stability of rice antioxidant system under salt treatment conditions. The activities of antioxidant enzymes were lower in KO lines and higher in OE lines compared with the WT rice (Fig. 9). Five *OsJRL45*-associated genes were identified by RNA-seq and interaction network analysis. Further analysis revealed that Os10g0210500 may be involved in the regulation of stress-responsive proteins, ion transporters and other proteins related to the salt stress response (Fig. 14). The RT-qPCR analysis showed that these *OsJRL45*-related genes were downregulated in KO mutants and upregulated in OE lines (Fig. 15). Therefore, we speculate that Os10g0210500 may be an important protein regulated by OsJRL45. This indicates that *OsJRL45* affects the salt tolerance of rice by regulating the expression of ion transport and other salt-stress responsive genes. Studies show that hormones play an important role in regulating the salt tolerance of plants [77]. The *NCED* family genes regulate salt tolerance in rice by regulating the synthesis of abscisic acid (ABA) [78]. Jasmonic acid (JA) enhances salt tolerance in wheat through a regulatory pathway of genes and enzymes related to the antioxidant system, plant hormones, and transcriptional regulation of physiological and biochemical processes [79]. Upregulation of auxin transporter gene expression under salt stress affects the salt tolerance of rice [80, 81]. The JRL family proteins are involved in the response to multiple plant hormones. For example, a *JRL* gene of Moso bamboo responds to a variety of hormones such as SA, ABA, and methyl jasmonate (MeJA) [47]; the *TaJRL1* gene of wheat is involved in the response to SA and MeJA [45]; a JRL protein of poplar mediates ABA response, maintaining antioxidant capacity and sodium-potassium balance under salinity treatment conditions [47]; and *OsJRL* regulates the response to ABA and affects the expression of genes associated with the ABA signaling pathway [49]. Our date suggested that *Os10g0210500* is involved in the hormone signaling pathway (Fig. 15). Auxin transporters PIN1A, PIN1C, and PIN2 participate in the auxin-activated signaling pathway, and simultaneously mediate the response to various hormones such as ABA, gibberellin (GA), and SA [63]. *CAX3* responds to ABA and ethylene [62]. *WOX4* mediates the response to GA, ABA, indole-3-acetic acid (IAA), and 6-benzylaminopurine (6-BA) [65]. The *TCP* family genes regulate plant hormones involved in salt stress [64, 82]. Thus, we infer that *OsJRL45* improves the salt tolerance of rice by participating in the regulation of plant hormones, in addition to regulating ion balance and improving antioxidant capacity. The specific hormone response mechanism of *OsJRL45* will be investigated in a future study.

SR86 is a semiwild salt-tolerant rice germplasm with a unique gene pool [83]. Mining salt-tolerant genes in SR86 and analyzing their physiological and molecular functions can provide theoretical guidance for developing new salt-tolerant rice resources. Some progress has been made in the whole-genome and transcriptome sequencing of SR86. Chen et al. found a large number of DEGs and salt stress-related genes in SR86 through GWAS and RNA-seq [36]. RNA-seq analysis of SR86 seedlings showed that *OsNACL35* improves salt tolerance by promoting the production of hydrogen sulfide (H_2_S) [84]. In this study, the salt tolerance-conferring gene *OsJRL45* was found in SR86 by BSA-seq analysis. The expression level of *OsJRL45* was significantly increased after salt treatments. *OsJRL45* significantly improved the salt tolerance of the salt-sensitive *indica* cultivar L6-23 and increased its grain yield after salt treatments (Figs. 7 and 8). The seed setting rate of Lh was 60% in soil containing 150 mM NaCl (Fig. 8). This shows that *OsJRL45* isolated from SR86 has unique characteristics and potential application in in salt-tolerant rice breeding. We aim to a conduct an in-depth interpretation of the salt tolerance molecular mechanism of *OsJRL45* in the future.

## Conclusions

In this study, we isolated the *JRL* family gene *OsJRL45* from SR86. *OsJRL45* was highly expressed in leaves, and the corresponding protein localized mainly to the endoplasmic reticulum. *OsJRL45* enhanced salt tolerance throughout rice the growth period. Overexpression of *OsJRL45* increased the 1000-grain weight and other agronomic traits of rice under salt treatments, and significantly improved rice yield. Additionally, *OsJRL45* enhanced the salt tolerance and yield of the salt-sensitive *indica* rice cultivar L6-23, revealing its potential in the breeding of salt-tolerant germplasm resources. Furthermore, *OsJRL45* enhanced the antioxidant capacity of rice and maintained Na^+^-K^+^ homeostasis under salt treatments. Finally, these results indicated that *OsJRL45* may affect the salt tolerance of rice by regulating Os10g021050 and affecting ion transporters and salt stress- and hormone response-related proteins.

## Methods

### Plant materials

‘Sea rice 86’ (SR86), a cultivar widely used in studies of rice salt tolerance [27, 38–40], was obtained by Rice germplasm resource platform of Hunan Rice Research Institute. Relevant data of the materials can be displalyed at https://www.ricedata.cn/variety/varis/618124.htm. Nipponbare (Nip), a cultivar of *japonica* rice, Chaling common wild rice (CLCWR) (*Oryza rufipogon* Griff.) and early *indica* rice Guangluai 4 (GLA4) were provided from Hunan Normal University, China. Rice variety were identified by the co-author, Associate Professor Dai Xiaojun. The L6-23 cultivar is a hybrid offspring of CLCWR and GLA4. All rice varieties were planted in the trial fields of Hunan Normal University, Changsha, Hunan Province, China. The experimental research reported here complies with institutional, national, and international guidelines concerning plant genetic repositories.

### Salt stress treatments

After germination, the seeds of SR86 and Nip were transplanted into conventional hydroponic medium for about 14 days, and these plants were grown to the trifoliate stage. These plants were then grown for 4 days in the presence of different concentrations of salt, then transferred to conventional hydroponic medium for 7 days, and the survival rate of the seedlings was finally determined. The F_2_ seeds obtained by crossing Nip and SR86 were cultured under 37°C illumination for 3 days. Seedlings with consistent growth were sown in the nursery. Fifty parental and five hundred F_2_ seedlings, with 3–4 tillers, were numbered, and each seedling was divided into two seedlings, one seedling was planted in salt-free soil, another seedling was planted in 150 mM salt soil. Under normal and salt treatment conditions, the phenotypes of rice plants during the whole growth stages were observed and recorded. 30 plants with extremely salt intolerance and extremely salt tolerant phenotypes were selected from the normal condition, and sent to Biomarker Technologies for BSA-seq.

Transgenic plants were generated using Nip and L6-23 as the WT backgrounds. The germinated seeds were incubated on Murashige and Skoog (MS) media containing different concentrations of salt and grown for 7 d. Then, root and shoot lengths of seedlings were determined. Rice seedlings were grown about 14 d under conventional hydroponic condition, then transferred to hydroponic conditions containing 100 mM salt concentration for 4 d, and finally transferred to conventional hydroponic medium for 5 d. The chlorophyll content, fresh weight, dry weight, and survival rate of rice plants were measured [73]. In order to analyze the salt tolerance of rice during the reproductive period, rice plants at tillering period were transplanted into soil containing 150 mM salt to grow to maturity and their agronomic traits were quantified.

### Generation of *Osjrl45* knockout (KO) mutant and *OsJRL45* overexpression (OE) lines

The CRISPR/Cas9 technology was executed to construct the knockout mutant plants. Reference to the standards of Maio et al. [85], the target site of 20 bp was selected (1: TCCGGCATCATCCTCGGCTG; 2: GGATGGCTCATCGATGCCAT). The recombinant plasmid was transformed into japonica rice Nip by *Agrobacterium* tumefaciens, and the transgenic plants were screened with hygromycin, then the specific primers (Supplementary Table 1) were used for PCR sequencing identification. We obtained two knockout mutants, KO1 and KO2, and off-target cleavage was detected using CRISPR-P (http://cbi.hzau.edu.cn/cgi-bin/CRISPR) [86]. To obtain the *OsJRL45* overexpression plants, the open reading frame (ORF) of *OsJRL45* was amplified from cDNA of Nip rice by PCR using the 1390-*OsJRL45*-F/-R primer (Supplementary Table 1), and the purified PCR product was restriction enzyme into pCAMBIA1390-Ubi plasmid with with *Spe*I and *Bam*HI. The promoter of *OsJRL45* was amplified from SR86 rice genomic DNA. The pCAMBIA1300 plasmid with *Bam*HI and *Hind*III restriction enzyme was used to clone into purified PCR products and transformed into the rice material L6-23 by *Agrobacterium* tumefaciens transformation. All recombinant and PCR products were verified by sequencing (Tsingke Biotech, Beijing).

### Gene expression analysis

The TRIzol Reagent (Invitrogen, Shanghai, USA) was used to extract total RNA of rice plants and the reverse transcription kit (Thermo Fisher Scientific, USA) was used to synthesize cDNA. The Takara Quantitative Kit RR420A (Takara, Dalian, China) was used to quantitative analysis. Then, The ABI PRISM 7500 Real-Time PCR instrument (Applied Biosystems) was used to perform RT-qPCR using primers (Supplementary Table 1). The 2^–△△CT^ method [66] was used to calculate expression values for each sample with *OsActin* gene as a reference value.

### GUS staining assay

To create the GUS transgenic plants, the *OsJRL45* promoter was cloned from Nip rice using 1301-*OsJRL45*-F/-R (Supplementary Table 1). As described by Hiei et al. [87], the amplified fragment was ligated into pCAMBIA1301 empty vector, the ligated recombinant plasmid was transferred into *Agrobacterium* EHA105. GUS staining experiments were carried out according to Li et al. [88]. Briefly, fresh harvested tissues of *pOsJRL45:* GUS transgenic mutant plants were collected in tube, to which 1 mL of GUS buffer and 10 µl of X-Gluc staining solution were added. The samples were placed in a 37°C water bath for 12 h. All samples were then destained with 75% alcohol until chlorotic. Finally, each sample was photographed with Olympus SZX7 camera.

### Subcellular localization analysis of *OsJRL45*

The *OsJRL45* CDS was duplicated from Nip rice DNA using 1390-GFP-F/R (Supplementary Table 1) and ligated into the pCAMBIA1390-GFP plasmids predigested with *Pst*I. The resulting 35S:*OsJRL45*-GFP recombinant was transiently transformed into Nip protoplasts via polyethylene glycol (PEG)-mediated transformation method. The endoplasmic reticulum Maker HDEL reported earlier was used [51, 52]. The Zeiss LSM 880 microscope was used to detect GFP signal [89].

### Measurement of chlorophyll content

To test the chlorophyll content of rice plants, leaves were cut from the rice seedlings and divided into small pieces. Then, the100 mg ground fresh leaves with 80% ice-cold acetone were used to extract chlorophyll, and a UV2400 UV/VIS spectrophotometer was used to measure the absorbance at 663 and 645 nm of each sample [78].

### Measurement of malondialdehyde (MDA) content and electrical conductivity

The MDA content was determined according to Heath et al. [90]. Fresh leaves (1 g) were ground on ice with trichloroacetic acid (TCA), the supernatant was separated by centrifugation and added to thiobarbituric acid (TBA), then the mixed solution was placed in a boiling water bath for about 15 min, the absorbance values at 450,532 and 600 nm after cooling to room temperature were determined respectively.

The relative conductivity is determined according to Gao et al. [91]. The conductivity meter was used to measure the electrical conductivity of the leachate from about 0.5 g of leaf segment organizations before (a1) and after the salt stress (a2). the absolute conductivity after boiling (a3) was measured.

### Evaluation of antioxidant enzyme activity and H2O2 content

The WT and knockout and overexpression of transgenic plants were grown in NaCl-free hydroponic medium about14 days and then grown in 100 mM salt-containing hydroponic solution about 2 days. Leaf samples (0.2g) were extracted by homogenizing them in phosphate buffer at 4℃.The kit instruction (Jiancheng Bioengineering Institute, Nanjing, China) was used to test the activities of SOD, POD and CAT enzymes and H_2_O_2_ content.

### Measurement of Na^+^ and K^+^ contents

The roots and shoots of WT, knockout and overexpression of transgenic plants were homogenized respectively and filtered with a sieve. Then, 50 mg of the ground sample was added to 2 ml of nitric acid (5): perchloric acid (1) mixture solution. The total volume of the sample was adjusted to 10 ml by addition of mixture solution. The inductively coupled plasma/optical emission spectrometry (Varian 715-ES) was used to measure the concentration of potassium and sodium ions in the sample after digestion and filtration.

### Transcriptome analysis

The whole plants of WT and KO were sampled before and after the salt treatments, and then sent to Biomarker Technologies for transcriptome analysis. Transcriptome data analysis was described previously [92].

### Interaction network analysis

Interaction network analysis was carried out according to Serin et al. [93–95]. Briefly, the DEG data were log-transformed and normalized, and then subjected to correlation analysis. Gene co-expression network analysis was obtained using the WGNCA and Cytoscape platforms. The String website (https://cn.string-db.org/) was used to perform Protein–protein interactions analysis.

### Statistical analysis

Data are showed as the mean ± standard deviation of three or more independent replicates. The PASW statistics 18 software was used to calculate the average of these replicates for each experiment, and One-way analysis of variance (ANOVA) and Student’s *t*-test at *P* < 0.05 were used to detect statistically significant differences. The GraphPad Prism 5 was used to constructed figures.

### Supplementary Information

**Additional file 1: Supplementary Table 1.** List of primers used in the study. **Supplementary Table 2.** List of salt-upregulated DEGs identified in KO. **Supplementary Table 3.** List of salt-downregulated DEGs identified in KO. **Supplementary Table 4.** The details of the plant materials.

## Data Availability

All of the data and statistics of this study can be found in the figures and tables of the manuscript. RNA sequencing data for rice is available in NCBI SRA database with accession numbers (SAMN37404049-SAMN37404060 of BioProject number PRJNA1017661, https://www.ncbi.nlm.nih.gov/bioproject/PRJNA1017661), and the data can be obtained with reasonable request of the corresponding author.
